# Suicide related ideation and behavior among Canadian gay and bisexual men: a syndemic analysis

**DOI:** 10.1186/s12889-015-1961-5

**Published:** 2015-07-02

**Authors:** Olivier Ferlatte, Joshun Dulai, Travis Salway Hottes, Terry Trussler, Rick Marchand

**Affiliations:** Faculty of Health Sciences, Simon Fraser University, Blusson Hall Room 11300, 8888 University Drive, Burnaby, BC V5A 1S6 Canada; Community-Based Research Centre for Gay Men’s Health, Vancouver, Canada; Dalla Lana School of Public Health, University of Toronto, Toronto, Canada

**Keywords:** Gay men, Bisexual men, Suicide, Syndemic, Homophobia, Violence, Canada

## Abstract

**Background:**

While several studies have demonstrated that gay and bisexual men are at increased risk of suicide less attention has been given to the processes that generate the inherent inequity with the mainstream population. This study tested whether syndemic theory can explain the excess suicide burden in a sample of Canadian gay and bisexual men. Syndemic theory accounts for co-occurring and mutually reinforcing epidemics suffered by vulnerable groups due to the effects of social marginalization.

**Methods:**

This study used data from Sex Now 2011, a cross-sectional survey of Canadian gay and bisexual men (*n* = 8382). The analysis measured the extent to which anti-gay marginalization and several psychosocial health problems are associated with suicide related ideation and attempts. Since psychosocial health problems were hypothesized to have an additive effect on suicide related ideation and attempts, the analysis calculated the effect of accumulated psychosocial health problems on suicide behavior.

**Results:**

Suicide ideation and attempts were positively associated with each individual marginalization indicator (verbal violence, physical violence, bullying, sexual violence and work discrimination) and psychosocial health problems (smoking, party drugs, depression, anxiety, STIs, HIV risk and HIV). Furthermore, prevalence of suicide ideation and attempts increased with each added psychosocial health problem. Those who reported 3 or more had 6.90 (5.47–8.70) times the odds of experiencing suicide ideation and 16.29 (9.82–27.02) times the odds of a suicide attempt compared to those with no psychosocial health problems.

**Conclusions:**

This investigation suggests that syndemics is a useful theory for studying suicide behavior among gay and bisexual men. Moreover, the findings highlight a need to address gay and bisexual men’s health problems holistically and the urgent need to reduce this population’s experience with marginalization and violence.

## Background

It has been well established that gay and bisexual men experience disproportionately higher rates of suicide related ideation and behavior. According to a meta-analysis conducted by King and his colleagues, gay and bisexual men are approximately four times more likely to attempt suicide over their lifetime in comparison to heterosexual men [1]. While evidence of this disparity has been accumulating, there has been less attention to the processes that have generated it [2].

Global theories of suicide, such as the interpersonal psychological theory [3] and clinical model [4], have been described as inadequate on their own for explaining the burden of suicide related ideation and behavior among gay and bisexual men [5]. Therefore, theories that explicitly take into account the unique experiences of sexual minorities are critical to better understanding the processes that sustain this inequity. Thus far, minority stress model [6], psychological mediation model [7] and ecological systems models [8] have emerged in the suicide literature as three theories that potentially address the unique vulnerabilities of sexual minorities to suicide related ideation and behavior, though few of these have been tested with empirical data [5, 9].

Another conceptual framework that may help explain the excess rate of suicide related ideation and behavior among gay and bisexual men may be found in syndemic theory. The term syndemics was coined by medical anthropologist Merrill Singer in the mid-90s to describe how health problems tend to co-occur, overlap and fuel each other to create a mutually reinforcing cluster of epidemics [10, 11]. More so, according to syndemic theory, these intersecting epidemics are understood to be the consequences of social inequity and the unjust exercise of power [11]. Syndemic theory distinguishes itself from other theories that link marginalization to negative health outcomes by its holistic lens - rather than seeing health outcomes, such as suicide and suicide related ideation and behavior, in isolation from other health problems, syndemic theory illuminates the synergistic interactions of multiple health problems and how these co-occurring epidemics affect communities and therefore degrade the overall health status of a population.

Syndemic theory has gained momentum in the gay and bisexual men’s health literature in recent years. It was first applied to gay and bisexual men by Stall and colleagues in a random sample survey of nearly 3000 gay and bisexual men in four major US cities. That study found that polydrug use, depression, childhood sexual abuse, and intimate partner violence were highly correlated among gay and bisexual men [12]. Furthermore, the study showed that accumulations of these health problems were significantly associated with sexual risk and HIV infection.

Since then several other public health studies have supported the existence of syndemics among gay and bisexual men. Stall, Friedman and Catania have also proposed a theoretical framework of syndemic production for gay men-informed by minority stress theory that posits that syndemics are socially produce by early adolescence anti-gay violence and stresses associated with migration to large cities to so called “gay ghettos” [13]. Most syndemic studies, including the theoretical framework of Stall and colleagues, have focused on HIV transmission risks, not suicide [14–24]. One notable exception is the work of Mustanski and colleagues who examined suicide among young gay and bisexual men. That study found that the experience of victimization increased syndemic burden. Syndemic conditions appeared to increase the odds of suicide attempts [9]. Those findings suggest that syndemic theory may be a potentially useful framework for the study of suicide among sexual minorities.

In this paper, the theory of syndemic will be applied to investigate suicide related ideation and behavior in a sample of Canadian gay and bisexual adult men. Drawing on the theoretical and empirical work above, the excess suicide related ideation and behavior was hypothesized to be socially produced through exposure to multiple forms of violence and highly correlated with other mental health and psychosocial problems common in gay and bisexual men’s lives. The ultimate goal is to understand the factors contributing to higher rates of suicide related ideation and behavior in gay and bisexual men, in order to generate strategies for suicide prevention specific to these groups.

## Methods

*Sex Now* is a serial cross-sectional survey of gay and bisexual men administered every 12–24 months since 2002 in the Canadian province of British Columbia. The survey has been offered anonymously online since 2007. The sampling frame was expanded to include all of Canada in 2010. This paper uses data from the 2011 edition of the survey collected from September 2011 to February 2012. The survey was offered in Canada’s two official languages, French and English. *Sex Now* 2011 was the first large-scale Canadian survey of gay men’s social determinants of health. The questionnaire was developed in collaboration with a group of young investigators, who participated in all phases of the study, including questionnaire development, pilot testing, recruitment, survey administration, data analysis, and communication of results (including the present report). The domains of *Sex Now* 2011 included: sexual behaviors, health measures, relationships, health care services, working conditions, community participation, social support, and experiences of homophobia.

Participants of *Sex Now 2011* were recruited from dating/sex-seeking websites (52.7 %), social media (23.1 %), an email database of previous survey participants (9.9 %), word of mouth (8.7 %), and other promotion activities (5.2 %). The survey protocol was reviewed by the independent Research Ethics Board of the Community-Based Research Centre for Gay Men’s Health.

### Measures

#### Demographic factors

Participants self-reported sexual orientation, partnerships status, education level, income, age, ethnicity, living environment and their province of territory of residence. All variables were categorical with the exception of age which was collected as a continuous variable.

#### Suicide related ideation and behavior

Two indicators of suicide related ideation and behavior were drawn from the following questions: a) “have you ever thought about suicide?” hereafter referred to as suicide ideation; and, b) “have you ever attempted suicide?” hereafter referred to as suicide attempt. In both cases, participants could report these as having never occurred, as having occurred prior to the last 12 months, within the last 12 months, or both prior to and within the last 12 months.

#### Marginalization indicators

Data on lifetime experiences of anti-gay marginalization and violence were collected. Participants were asked if they had experienced: a) verbal violence and/or hate talk, b) physical violence, c) anti-gay bullying (i.e. harassment, cyber-bullying) d) sexual violence (i.e. unwanted sex), and e) workplace discrimination based on their sexuality.

#### Psychosocial health problems

Participants were asked if they had experienced the following in the last 12 months: a) frequent consumption of tobacco (regular/daily smokers), b) use of one or multiple of the following party drugs: cocaine, crystal meth, ecstasy, GHB and ketamine, c) being on medication for depression, d) being on medication for anxiety, e) being diagnosed with one or more of the following sexually transmitted infections (STIs): gonorrhea, chlamydia, syphilis, herpes, HPV or hepatitis C, f) one or more episodes of condomless anal intercourse (insertive or receptive) with a partner (CAI-US), whose HIV status was unknown or discordant. We also asked participants to report whether g) they have ever been diagnosed with HIV.

### Analytic plan

**Fig. 1:**
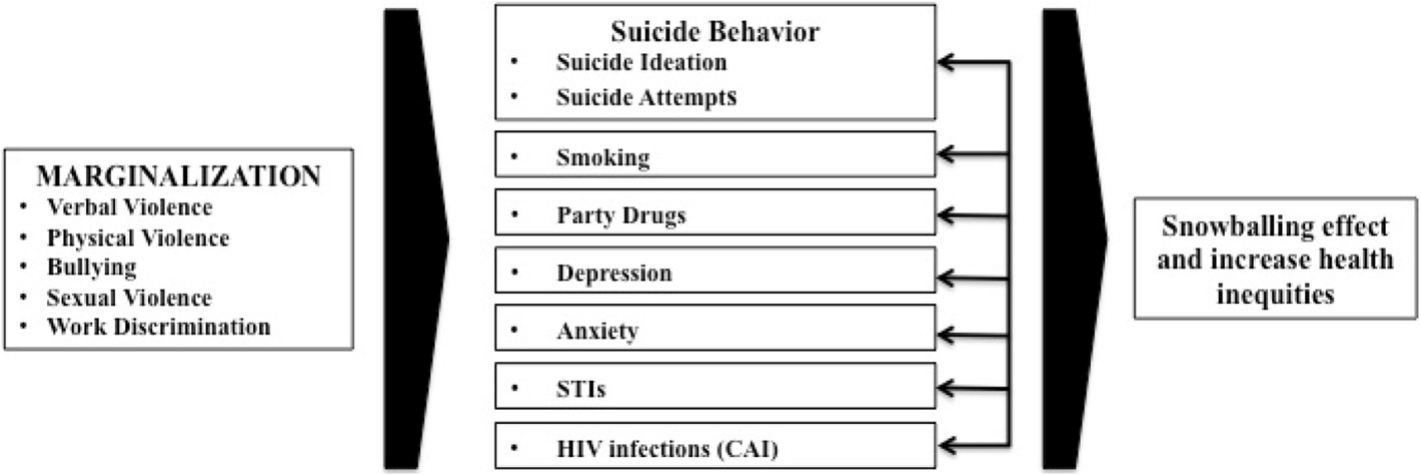
Conceptual Framework of Suicide Risk within Syndemic Production

The analysis was guided by the syndemic production theory of Stall, Friedman and Cantania [13]. Consistent with this theory and previous syndemic studies [11, 17, 18], the accumulation of social stressors was hypothesized to lead to the development of psychosocial health problems, including suicide ideation and attempts. These health problems were predicted to be interrelated and mutually reinforcing (independently associated) -a “snowball” effect in syndemic theory (as described in Fig. 1). The analytic plan for this study was modeled on a syndemic-based analysis of HIV transmission risk in the 2010 version of the *Sex Now* survey [24].

First the relationships between lifetime indicators of anti-gay marginalization, suicide ideation and attempted suicide in the last 12 months were explored using logistic regression. Because the effect of marginalization was hypothesized to be additive, we calculated the percentage of respondents who experienced suicide ideation and who attempted suicide in the last 12 months by number of marginalization indicators. These relationships were tested using Chi-Square test for trend with p < 0.05 considered statistically significant.

Second, because suicide related ideation and attempt were hypothesized to be interrelated with and to have mutually reinforcing relationships with other psychosocial health problems, we examined correlations between all psychosocial health problems, including suicide ideation and suicide attempts in the last 12 months. Since those who experience multiple psychosocial health problems (those caught in syndemics) were hypothesized to be at increased risk for suicide ideation and attempts, the percentage of those who reported suicide ideation and attempted suicide in the last 12 months was calculated for each expanding number of psychosocial health problems.

Lastly, associations between individual psychosocial health problems and the count of those problems associated with suicide ideation or having attempted suicide were explored in logistic regression.

All multivariable models were adjusted for demographic variables including sexual orientation, as some researchers have found differences in suicide related ideation and behavior between gay and bisexual men [25]. Ninety-five percent confidence intervals (CIs) were calculated for all odds ratios; 95 % CIs which excluded 1 (*p* < 0.05) were considered statistically significant, though in interpreting results, emphasis is placed on the magnitude of effect. All analyses were performed using SPSS 20.0.

## Results

8382 Canadian men participated in *Sex Now 2011* and all were included in the analysis. The demographic characteristics of the sample are described in Table 1. Among participants, 49.9 % (*n* = 4,180) reported lifetime experience of suicide ideation, while 12.5 % (*n* = 1,050) reported having attempted suicide in their lifetime. When asked about these experiences in the last 12 months, 17.0 % (*n* = 1,427) reported experiencing suicide ideation, while 1.7 % (*n* = 145) reported a suicide attempt. 63.13 % (*n* = 901) of those who reported suicide ideation in the last year reported similar experiences over a year ago, while 71.7 % (104) of those who attempted suicide in the last 12 months also reported a previous attempt over a year ago.

**Table 1:** Characteristics of study participants (*N* = 8382)

Characteristics	No. (%)
Sexual orientation	
Gay	5406 (64.5)
Bisexual	2720 (32.5)
Straight	175 (2.1)
Other	81 (1.0)
Relationship status	
Single	3675 (43.8)
Partnered with a man	2215 (26.4)
Partnered with a woman	1798 (21.4)
Divorced/Separated/Other	694 (8.3)
Age	
Under 30	1866 (22.43)
30–45 years old	2518 (30.0)
Over 45	3998 (47.7)
Ethnicity	
Caucasian	7310 (87.2)
Asian	212 (2.5)
South Asian	74 (0.9)
African/Caribbean	76 (0.9)
Latino/Hispanic	112 (1.3)
Aboriginal (First Nation/Inuit/Metis)	168 (2.0)
Middle Eastern	53 (0.6)
Mixed	229 (2.7)
Other	136 (1.6)
Income (annual, CAD)	
<10,000	739 (8.8)
10,000–29,9999	1653 (19.7)
30,000–49,9999	1847 (22.0)
50,000–69,0000	1698 (20.2)
>70,0000	2444 (29.2)
Highest level of Education Completed	
Some high School	348 (4.2)
High School	1131 (13.5)
Some College or University	2116 (25.2)
College	1607 (19.2)
University	3180 (37.9)
Province/Territories	
British Columbia	1804 (21.5)
Alberta	1064 (12.7)
Saskatchewan	289 (3.4)
Manitoba	342 (4.1)
Ontario	3366 (40.2)
Quebec	1048 (12.5)
New-Brunswick	105 (1.3)
Nova Scotia	223 (2.7)
Prince Edward Island	33 (0.4)
Newfoundland and Labrador	85 (1.0)
Yukon	8 (0.1)
Norwest Territories	11 (0.1)
Nunavut	4 (0.0)

**Fig. 2:**
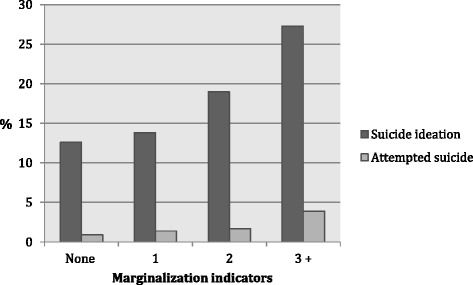
Prevalence of suicide related ideation or attempts by number of anti-gay marginalization indicators

Table 2 shows the bivariate relationships between anti-gay marginalization indicators and suicide ideation or having attempted suicide in the last 12 months. All indicators were positively associated and statistically significant (*p* < 0.05) when adjusted for demographics (OR range from 1.30 to 3.94). The effects of all variables diminished in the multi-indicator model (i.e. with adjustment for all indicators of marginalization), though most remained statistically significant with odds ratios in the range of 1.30 to 2.81, among those which remained statistically significant. When adjusted for the other forms of marginalization, the associations between sexual violence and suicide ideation and bullying and suicide attempts were no longer statistically significant, and the association between verbal violence and suicide attempts was removed. The cumulative effects of marginalization, suicide ideation and having attempted suicide in the prior 12 months are presented in Fig. 2. For each additional form of marginalization reported, there was an increased risk of both ideation and attempted suicide (Chi-square test for trend p < .001). People who reported three or more forms of anti-gay marginalization had twice the rate of suicide ideation compared to those with no marginalization (27.3 % vs 12.6 %), and over 4 times the rate of attempted suicide (3.9 % vs 0.9 %).

**Table 2:** Correlation between anti-gay marginalization and suicide related ideation and attempts (*N* = 8382)

		% reporting suicide ideation or attempts			
	No. (%) reporting marginalization indicator	Among those with indicator	Among those without indicator	Single indicator models AOR (95 % CI)^a^	Multi-indicator model AOR (95 % CI)^b^
Suicide ideation in the last 12 months					
Verbal Violence	3920 (46.8 %)	21.6 %	13.0 %	1.77 (1.55–2.01)	1.30 (1.11–1.51)
Physical Violence	1044 (12.5 %)	27.0 %	15.6 %	1.90 (1.62–2.21)	1.36 (1.14–1.61)
Bullying	3495 (41.7 %)	22.1 %	13.4 %	1.73 (1.53–2.00)	1.30 (1.12–1.50)
Sexual Violence	985 (11.8 %)	20.1 %	16.6 %	1.30 (1.10–155)	1.13 (0.95–1.35)
Work Discrimination	1353 (16.1 %)	25.3 %	15.4 %	1.91 (1.65–2.20)	1.47 (1.26–1.71)
Attempted suicide in the last 12 months					
Verbal Violence	3920 (46.8 %)	2.3 %	1.2 %	1.95 (1.35–2.83)	0.95 (0.60–1.53)
Physical Violence	1044 (12.5 %)	5.3 %	1.3 %	3.94 (2.76–5.61)	2.81 (1.84–4.31)
Bullying	3495 (41.7 %)	2.5 %	1.2 %	2.06 (1.44–3.00)	1.24 (0.80–1.92)
Sexual Violence	985 (11.8 %)	4.1 %	1.4 %	2.92 (2.01–4.26)	2.20 (1.48–3.25)
Work Discrimination	1353 (16.1 %)	3.5 %	1.4 %	2.71 (1.88–3.90)	1.63 (1.08–2.45)

^a^Adjusted for age, ethnicity, income, education, sexual orientation, living environment and province of residences

^b^Adjusted for the demographic variable above and all other marginalization indicators

Table 3 shows that all psychosocial health problems were positively associated and statistically significant (p < .005) with the marginalization indicators at the exception of STIs with sexual violence. The correlations between psychosocial health problems are presented in Table 4; all were correlated with the exception of smoking and STI (which did not achieve statistical significance at *p* < 0.05). 76.6 % (*n* = 111) of the 145 men who attempted suicide in the last 12 months reported at least one other health problem while 38 % (*n* = 55) reported 3 or more psychosocial health problems. Similarly, 66.4 % (*n* = 948) of the men who reported suicide ideation reported at least one co-occurring issue in the prior 12 months, while 15.1 % (*n* = 216) reported three or more.

**Table 3:** Correlation between marginalization and psychosocial and health issues

		Unadjusted Odds Ratio (95 % Confidence Interval)				
	N (%)	Verbal Violence	Physical Violence	Bullying	Sexual Violence	Work Discrimination
Smoking	2061 (24.6)	1.20 (1.09–1.33)	1.52 (1.32–1.75)	1.19 (1.08–1.31)	1.68 (1.46–1.94)	1.24 (1.09–1.41)
Party Drugs	1279 (15.3)	2.24 (1.98–2.54)	2.28 (1.96–2.66)	2.00 (1.77–2.56)	1.61 (1.37–1.91)	1.50 (1.29–1.74)
Depression	945 (11.3)	1.88 (1.65–2.14)	1.97 (1.67–2.32)	1.83 (1.61–2.09)	1.41 (1.18–1.69)	1.90 (1.63–2.21)
Anxiety	1081 (12.9)	2.05 (1.78–2.36)	2.26 (1.91–2.68)	2.09 (1.82–2.40)	1.48 (1.22–1.79)	2.13 (1.82–2.50)
STIs	661 (7.9)	1.41 (1.20–1.65)	1.80 (1.47–2.21)	1.40 (1.20–1.65)	1.17 (0.92–1.48)	1.59 (1.31–1.93)
CAI–US	2515 (30.0)	1.47 (1.34–1.62)	1.65 (1.44–1.88)	1.43 (1.31–1.58)	1.57 (1.37–1.80)	1.59 (1.41–1.80)
HIV Positive	667 (8.0)	2.56 (2.16–3.02)	2.58 (2.14–3.12)	2.04 (1.73–2.39)	1.94 (1.58–2.38)	2.71 (2.27–3.22)

**Table 4:** Correlation between psychosocial and health issues

		Unadjusted Odds Ratio (95 % Confidence Interval)					
	N (%)	Party Drugs	Depression	Anxiety	STIs	CAI-US	HIV Positive
Smoking	2061 (24.6)	2.47 (2.18–2.80)	1.39 (1.21–1.60)	1.51 (1.30–1.75)	1.08 (0.90–1.29)	1.14 (1.03–1.27)	1.26 (1.06–1.51)
Party Drugs	1279 (15.3)		1.38 (1.17–1.62)	1.58 (1.33–1.87)	2.87 (2.41–3.43)	2.75 (2.43–3.11)	3.45 (2.91–4.10)
Depression	945 (11.3)			29.28 (24.84–54 .50)	1.76 (1.44–2.16)	1.35 (1.18–1.54)	2.49 (2.06–3.00)
Anxiety	1081 (12.9)				1.88 (1.52–2.31)	1.18 (1.02–1.36)	1.92 (1.56–236)
STIs	661 (7.9)					2.58 (2.20–3.03)	3.96 (3.23–4.86)
CAI-US	2515 (30.0)						3.48 (2.96–4.09)
HIV Positive	667 (8.0)						

The associations between psychosocial health problems and suicide ideation and attempts are presented in Table 5**.** While all associations but one were statistically significant in single indicator models, when entered into a multiple indicator model only some associations remained significant. For suicide ideation, depression [AOR 5.37 95 % CI 4.44 -6.49], anxiety [AOR 1.56 (1.27–1.92)], and sexual risk (CAI-US) [AOR 1.36 (95 % CI 1.18–1.57)] remained significant while for attempted suicide only depression [AOR 4.73 (95 % CI 2.84–7.88)] and anxiety [AOR 2.79 (95 % CI 1.67–4.65)] were significant.

**Table 5:** Association between psychosocial and health issues and suicide related ideation and attempts

		% with psychosocial or health issue			
	No. (%) reporting health Issues	Among those reporting suicide behavior	Among those not reporting suicide behavior	Single indicator models AOR (95 % CI)^a^	Multi-indicator model AOR (95 % CI)^b^
Suicide ideation in the last 12 months					
Smoking	2061 (24.6 %)	28.5 %	23.8 %	1.20 (1.04–1.38)	1.06 (0.91–1.24)
Party Drugs	1279 (15.3 %)	20.7 %	14.1 %	1.38 (1.17–1.62)	1.19 (0.99–1.43)
Depression	1081 (12.9 %)	36.6 %	8.0 %	6.87 (5.88–8.02)	5.37 (4.44–6.49)
Anxiety	945 (11.3 %)	27.8 %	7.9 %	4.22 (3.39–4.96)	1.56 (1.27–1.92)
STIs	661 (7.9 %)	10.6 %	7.3 %	1.42 (1.15–1.75)	1.11 (0.88–1.40)
CAI-US	2515 (30 %)	37.6 %	28.4 %	1.45 (1.27–1.65)	1.36 (1.18–1.57)
HIV Positive	667 (8.0 %)	7.5 %	10.2 %	1.37 (1.10–1.70)	0.94 (0.74–1.20)
Attempted Suicide in the last 12 months					
Smoking	2061 (24.6 %)	37.2 %	24.4 %	1.62 (1.10–2.38)	1.27 (0.84–1.92)
Party Drugs	1279 (15.3 %)	29 %	15 %	1.95 (1.29–2.94)	1.51 (0.94–2.41)
Depression	1081 (12.9 %)	55.9 %	12.1 %	9.13 (6.22–13.39)	4.73 (2.84–7.88)
Anxiety	945 (11.3 %)	49.7 %	10.6 %	8.05 (5.50–11.79)	2.79 (1.67–4.65)
STIs	661 (7.9 %)	16.6 %	7.7 %	2.21 (1.34–3.65)	1.68 (0.96–2.92)
CA-US	2515 (30 %)	40.0 %	29.8 %	1.51 (1.04–2.19)	1.27 (0.85–1.91)
HIV Positive	667 (8.0 %)	7.9 %	9.7 %	0.97 (0.51–1.87)	0.57 (0.28–1.16)

^a^Adjusted for age, ethnicity, income, education, sexual orientation, partnership status, living environment and province of residence

^b^Adjusted for the demographic variable above and all other health issues

Models in Table 6 demonstrate the additive relationship between psychosocial health problems and suicide related ideation and suicide attempts. Compared to those reporting no problems, those reporting one [AOR 1.69 (95 % CI 1.44–1.97)], two, [AOR 3.36 (1.44–1.97)] and three or more issues [AOR 6.90 (95 % CI 6.90–5.47–8.70)] were at increased odds of reporting suicide ideation. Similarly, those experiencing one [AOR 1.25 (0.70–2.23)], two [AOR 3.67 (95 % CI 2.15–6.28)] and three or more issues [AOR 16.29 (95 % CI 9.82–27.02)] were at increased odds of attempted suicide. The same trends were found, though the magnitude of ORs decreased and their confidence intervals overlapped, when we removed the two psychosocial health problems (depression and anxiety) which had the strongest individual effect on attempted suicide (data not shown). The AOR for continuous count of psychosocial health problems was 1.88 (95 % CI 1.76–2.00) for suicide ideation and 2.63 (95 % 2.20–3.14) for attempted suicide.

**Table 6:** Association between the cumulative number of psychosocial issues and suicide related ideation and behavior

	Number	Percent	AOR (95 % CI)^a^
Suicide ideation in the last 12 months^b^			
No issue	4465	53.3	Reference
One	2372	28.3	1.69 (1.44–1.97)
Two	1098	13.1	3.36 (2.82–4.01)
Three or More	447	5.3	6.90 (5.47–8.70)
Attempted suicide in the last 12 months^b^			
No issue	4465	53.3	Reference
One	2372	28.3	1.25 (0.70–2.23)
Two	1098	13.1	3.67 (2.15–6.28)
Three or More	447	5.3	16.29 (9.82–27.02)

^**a**^ Model adjusted for age, ethnicity, income, education, sexual orientation, partnership status, living environment and province of residence

^**b**^ Cumulative effect of depression, anxiety, smoking, party drugs, and STIs

## Discussion

Our results add further evidence to the existing literature that shows that gay and bisexual are at increased risk of suicide related ideation and behavior as compared with heterosexual men. Lifetime experience of suicide related ideation and behavior was reported by 49.9 % of our sample; this is over 6 times what has been reported among Canadian heterosexual men (7.4 %) [26]. More so, findings of this investigation strongly suggest that experiences of anti-gay violence and marginalization increase suicide ideation and suicide attempts among Canadian gay and bisexual men. Moreover, health problems in our sample were associated with each other and with suicide behavior. This study therefore adds to the growing body of literature showing that health disparities occur in clusters among gay and bisexual men. These results, like those of Mustanski [9], support the notion that syndemic theory has the potential to advance research, theory, and interventions related to suicide in this population.

There are some limitations that need consideration when interpreting these results. First of all, since the study relies on an Internet convenience sample it is difficult to know how representative it is of the Canadian gay and bisexual men’s population. Moreover, because the survey was cross-sectional and reliant on self-reported retrospective data, conclusions cannot be drawn about the causality of outcomes, including the relation between marginalization and negative health outcomes. Because these outcomes (both psycho-social mediators and suicide-related outcomes) were measured as currently prevalent (within last 12-months), we may have underestimated the true burden of these issues resulting from early-life marginalization experiences (e.g., if some respondents experienced depression or suicide ideation/attempts after encountering antigay bullying/harassment during adolescence but then adapted and recovered from these mental health conditions). This misclassification of psychosocial and suicide-related outcomes would result in *under*-estimation of the associations we present. More so, we modeled our analysis according to Stall’s theory of syndemic production [13] but were unable to account for all the factors that may produce or mitigate negative health outcomes such as internalized stigma and protective factors such a social support. We also combined gay and bisexual men in our analysis; however, experiences of oppressions and syndemics may differ between these groups and should be investigated in future studies. Finally, the cross-sectional survey involved people who had attempted suicide and survived. They may have somewhat different characteristics than those whose suicide attempt was fatal.

This paper adds to the plea of other syndemic researchers, as well as that of community activists, to address gay and bisexual men’s health holistically and to create better living conditions for sexual minorities. Since many of the health disparities experienced by gay and bisexual men (such as HIV, depression, anxiety, polydrug use, suicide) happen synergistically, it is unlikely that any of these conditions would improve without attending to the others. This is concerning in the current context where HIV and STIs are generally the health issues of gay and bisexual men that receive the most public health attention, while other issues, particularly those related to mental wellbeing, are largely ignored. Suicide prevention is notably absent from gay and bisexual public health and community programming.

In exploring the particular psychosocial health problems that constitute this syndemic, we found that each was associated with suicide related ideation and behavior, but the largest effect was found in evaluating the cumulative number of problems—as predicted by syndemic theory. It is striking that men who experienced three or more psychosocial health problems had approximately seven times the odds of suicide ideation and sixteen times the odds of attempted suicide, compared to those with no problems. When all health problems were included in a single model (multi-indicator), the association with suicide ideation and attempts was removed for most but remained strongest for depression. This should not be surprising, considering that mood disorders are known to convey the highest risk for suicide related ideation and behavior in general populations [27].

This study helps identify some of the potential root causes of suicide related ideation and behavior among gay and bisexual men. The five marginalization indicators investigated were positively associated with suicide ideation and behavior in our sample, and the majority of gay and bisexual men (56.1 %) experienced at least one form of marginalization or anti-gay violence in their lifetime. A previous edition of the *Sex Now* survey (2010) showed that prevalence of physical violence, sexual assault and workplace discrimination was similar across all age cohorts, but the younger cohort experienced higher levels of sexist harassment than any previous generation [24]. Some of the marginalization indicators this study examined (i.e., physical violence, harassment) were also found associated with suicide related ideation and behavior in previous studies [9]. Workplace discrimination was, however, a novel contribution of this study.

Together these findings suggest that, despite the recent legal gains such as the availability of gay marriage, adoption and protection from discrimination under the constitution, a substantial number of gay and bisexual men still face homophobia. This is consistent with other data sources such as Statistics Canada research which shows that while hate crimes appear to be on the decline in Canada in general, it is not the case for hate crimes committed against sexual minorities [28]. In addition, hate crimes committed against sexual minorities are more likely to be violent and to result in injuries. Another study of Canadian youth found that 20.4 % s of sexual minority men were verbally harassed weekly because of their sexuality, while 17 % reported being physical assaulted at school [29]. These trends are concerning as those who experienced physical violence had nearly 3 times the odds of attempted suicide in the survey.

Efforts to prevent physical assault against gay and bisexual men, as well as other forms of violence and discrimination, are therefore urgently needed to ameliorate the social and health condition of this population, including reducing the incidence of suicide attempts. However, policies and interventions can be made to increase the safety of sexual minorities. For example a school-based survey in the Canadian province of British Columbia demonstrated that gay and bisexual students attending schools with anti-homophobia policies and gay-straight alliances (GSAs) had lower odds of discrimination, as well as lower odds of suicide ideation and attempts [30].

## Conclusions

This study’s findings suggest that syndemics, that include suicide related ideation and attempts, are occurring among Canadian gay and bisexual men. This study also showed that psychosocial health problems have an additive effect on suicide, increasing the odds of suicide ideation and suicide attempts. Those who reported 3 or more psychosocial health problems had 6.9 times the odds of suicide ideation and 16.29 times the odds of a suicide attempt compared to those with no problems and these effects are driven in particular by experiences of depression and anxiety. These results highlight the importance of addressing gay and bisexual men’s health problems in a holistic manner rather than addressing each in isolation. Since all marginalization indicators increased the odds of suicide ideation and attempts, interventions to reduce social exclusion and violence are urgently needed.

These results concur with those of Mustanksi and colleagues which demonstrated that syndemic is a useful theory to advance understanding of suicide among gay and bisexual men. While both studies illuminate some of the pathways and social factors that produce suicide related ideation and behavior in this population, such influences have generally been inadequately attended to in research and remain poorly understood. Therefore suicide researchers ought to pay more attention to the pathways of suicide ideation and behavior, as they may be critical to the development of effective prevention interventions. Since the majority of gay and bisexual men experienced violence in our survey but only a small minority reported syndemics, suicide ideation and suicide attempts, an array of protective factors may exist. Identifying those would also be key to prevention and health promotion efforts. Furthermore, as the population of gay and bisexual men is diverse along axes of class, geography, education attainment, and ethnicity, researchers should also look at how suicide is experienced and distributed among differently positioned gay and bisexual men.
